# Systematic Genomic Surveillance of SARS-CoV-2 at Xiamen International Airport and the Port of Xiamen Reveals the Importance of Incoming Travelers in Lineage Diversity

**DOI:** 10.3390/v16010132

**Published:** 2024-01-17

**Authors:** Ruiluan You, Ruotong Wu, Xijing Wang, Rao Fu, Ningshao Xia, Yixin Chen, Kunyu Yang, Junyu Chen

**Affiliations:** 1 Xiamen International Travel Healthcare Center, Xiamen Entry-Exit Inspection and Quarantine Bureau, Xiamen 361001, China; youruiluan@163.com; 2 State Key Laboratory of Vaccines for Infectious Diseases, Xiang An Biomedicine Laboratory, Department of Laboratory Medicine, School of Public Health & School of Life Sciences, Xiamen University, Xiamen 361102, China; 21620221153502@xmu.edu.cn (R.W.); nsxia@xmu.edu.cn (N.X.); yxchen2008@xmu.edu.cn (Y.C.); 3 National Institute of Diagnostics and Vaccine Development in Infectious Diseases, State Key Laboratory of Molecular Vaccinology and Molecular Diagnostics, Collaborative Innovation Center of Biologic Products, National Innovation Platform for Industry-Education Integration in Vaccine Research, Xiamen University, Xiamen 361102, China

**Keywords:** border screening, SARS-CoV-2 variants, sentinel surveillance

## Abstract

Sever Acute Respiratory Syndrome Coronavirus-2 (SARS-CoV-2) is still a threat to human health globally despite the World Health Organization (WHO) announcing the end of the COVID-19 pandemic. Continued surveillance of SARS-CoV-2 at national borders would be helpful in understanding the epidemics of novel imported variants and updating local strategies for disease prevention and treatment. This study focuses on the surveillance of imported SARS-CoV-2 variants among travelers entering Xiamen International Airport and the Port of Xiamen from February to August 2023. A total of 97 imported SARS-CoV-2 sequences among travelers from 223 cases collected from 12 different countries and regions were identified by real-time RT-PCR. Next-generation sequencing was used to generate high-quality complete sequences for phylogenetic and population dynamic analysis. The study revealed a dominant shift in variant distribution, in which the XBB subvariant (XBB.1.5, XBB.1.16, XBB.1.9, XBB.2.3, and EG.5.1) accounted for approximately 88.8% of the sequenced samples. In detail, clades 23D and 23E accounted for 26.2% and 21.4% of the sequenced samples, respectively, while clades 23B (13.6%) and 23F (10.7%) took the third and fourth spots in the order of imported sequences, respectively. Additionally, the XBB.2.3 variants were first identified in imported cases from the mainland of Xiamen, China on 27 February 2023. The spatiotemporal analyses of recent viral genome sequences from a limited number of travelers into Xiamen provide valuable insights into the situation surrounding SARS-CoV-2 and highlight the importance of sentinel surveillance of SARS-CoV-2 variants in the national border screening of incoming travelers, which serves as an early warning system for the presence of highly transmissible circulating SARS-CoV-2 lineages.

## 1. Introduction

SARS-CoV-2 is still circulating worldwide and remains a global concern [1,2]. It appears that COVID-19 epidemics in the future will follow a year-round recurring mini-waves pattern rather than seasonal surges observed with influenza viruses, posing a continuing threat. SARS-CoV-2 is an RNA virus with a positive-sense, single-stranded ribonucleic acid. Typically, it undergoes one to two single-nucleotide mutations each month. Due to its widespread and continuous evolution, various variants of concern (VOCs) have emerged worldwide, including the Alpha (B.1.1.7), Beta (B.1.351), Gamma (P.1), Delta (B.1.617.2), and Omicron (B.1.1.529 and XBB.1.5) variants, causing multiple waves [3,4,5,6,7]. In particular, the XBB.1.5 subvariant significantly evades humoral immunity induced by vaccination and natural infection and has now replaced previously dominant Omicron variants globally [7,8]. Since its first detection in late 2022, it has rapidly become the prevailing strain. The emergence of numerous escape variants indicates that COVID-19 will persist in the human population for years to come. Therefore, real-time whole-genome sequencing is essential in providing valuable insights into the transmission dynamics of the pandemic and enables effective surveillance [9,10]. Furthermore, genomic data offer useful information necessary for the ongoing development of vaccines, therapeutics, and diagnostic tools [11]. Analysis of SARS-CoV-2 mutations is particularly crucial, especially when they affect epitopes responsible for inducing host immune responses, as they may lead to immune evasion, potentially affecting the efficacy of vaccines and immunotherapy.

China implemented a distinct outbreak response strategy prior to December 2022, utilizing precise prevention and control measures to halt the transmission of SARS-CoV-2 [12,13]. With the strategy called “Dynamic COVID-zero”, China took precise prevention and control measures to quickly find, control, and cure infected people in each cluster outbreak within a specific geographic region to avoid affecting social and economic development in other regions, so as to achieve the maximum effect at the lowest cost, especially for travelers returning from abroad. Due to the reduced pathogenicity of Omicron subvariants [14], widespread vaccination efforts, and the accumulation of experience in prevention and control, China further refined its strategies for managing COVID-19 in mid-November 2022. Due to the adjusted strategies for the prevention and control of SARS-CoV-2 transmission, quarantine is no longer mandatory for infected individuals, allowing them access to public areas. This policy change has led to a gradual increase in the number of infections across mainland China. Therefore, conducting a comprehensive spatiotemporal study on the circulating SARS-CoV-2 variants is of utmost importance in China to facilitate a global response to the ongoing COVID-19 epidemics.

Xiamen, a renowned tourist port city situated on the southeast coast of China adjacent to the Taiwan Strait, has experienced an increasing influx of international travelers following policy adjustments in China [15]. This has raised the risk of exposure to imported SARS-CoV-2 within the area. The significance of border screening in understanding the cross-border transmission of infectious diseases through imported cases has been underscored by the experiences gained during the influenza A (H1N1) pdm09 virus pandemics and the ongoing COVID-19 pandemic [16,17]. Therefore, the importance of long-term and continuous monitoring of the genomic profile of internationally circulating SARS-CoV-2 variants carried by incoming travelers at Xiamen International Airport and the Port of Xiamen, following the aforementioned policy adjustments, cannot be overstated. In this study, we employed genomic analysis based on entry border screening for SARS-CoV-2 variants in incoming travelers at Xiamen International Airport and the Port of Xiamen to describe the introduction and subsequent dispersal of the XBB lineages and its sublineages. Notably, our findings highlight a transition from the previously dominant XBB.2.3 and XBB.1.9 lineages to a new lineage, EG.5, which has been prevalent in China and globally since August 2023 following the detection of the first XBB.2.3 case among travelers arriving in Xiamen. By combining the collected genomic sequence data with individual travel histories, we performed travel-history-aware phylogeographic inference and identified slight differences in the lineage distribution between local and imported infections in Xiamen from February to August. Our inference suggests that the introduction of SARS-CoV-2 variants into Xiamen was closely associated with the local circulation of SARS-CoV-2 variants. Given the importance of these findings for regional SARS-CoV-2 surveillance, we emphasize the need to strengthen sentinel surveillance at the country’s points of entry.

## 2. Materials and Methods

### 2.1. Sample and Data Source

In-house samples for this study were collected at the Xiamen entry border, including airports and ports, from February to August 2023. All passengers and crew members with ILI (influenza-like illness) symptoms, screened by an infrared thermometer with a body temperature > 37.5 °C or with the obvious presence of specific symptoms, including cough, rhinorrhea, sore throat, and/or gastrointestinal disturbance, were required to be sampled at both the airports and ports. In addition, random sampling was conducted at a rate of 2–5% at the airports and ports for other passengers and crew members. All participants were invited to participate in a consultation to obtain their informed consent, basic personal information, and medical history. Nasopharyngeal swabs were placed in virus transport media immediately upon collection. Samples were extracted using an automated nucleic acid extractor (NFAST-32, Shenzhen United Medicine, Shenzhen, China), and the presence of SARS-CoV-2 RNA was detected by RT-qPCR assay on a BIORAD CFX96 DeepWell with a commercial test (DAAN Gene^®^, Zhongshan, China) using the CDC China protocol. A total of 100 samples with CT values less than 32 were selected for next-generation sequencing. Additionally, 166 SARS-CoV-2 genomic sequences and corresponding patients’ metadata from Xiamen, reported between February 2023 and August 2023, and 35 reference sequences were retrieved from GISAID (Supplementary File). The reference sequences were selected as representatives of the global SARS-CoV-2 variants covered from clade 19A to 23F. All of the genomic data were combined and filtered based on the sequencing quality. Among the 166 positive cases, 2 local sequences were eliminated after evaluation of the quality of the full genomes of low quality. Finally, a total of 299 sequences were processed. Travelers did not receive any kind of medical certificate, diagnosis, or therapeutic intervention in this study. This study was approved by the medical ethics committee at Xiamen University, China (XMULAC20200232).

### 2.2. Next-Generation Sequencing

SARS-CoV-2-positive samples were processed for NGS using the multiplexed PCR amplicon approach. In detail, viral RNA was extracted from a 200 μL sample by an NFAST-32 nucleic acid extraction system (Shenzhen unimed-global technology, Shenzhen, China). A SARS-CoV-2 whole-genome multiplex PCR product was generated from viral RNA using an ATOPlex RNA multiplex-PCR-based library preparation set (MGI Tech, Shenzhen, China), which combines the reverse transcription module and the multiplex PCR amplification module. The cDNA generation and multiplex PCR were performed on a TC1000-G gradient PCR instrument (Dlab scientific, Beijing, China). PCR product purification was performed and fast PCR-free libraries were prepared using an MGISP100 Automated Sample Preparation System (MGI Tech, Shenzhen, China). Afterward, the quantification of these libraries was tested on a Qubit 3.0 Fluorometer (Life Technologies, New York, NY, USA). Then, DNB (DNA Nanoballs) libraries were generated using a DNBSEQ one-step DNB preparation set (MGI Tech, Shenzhen, China) following the manufacturer’s instructions, and the sequencing was carried out on an MGISEQ-200 platform. Raw data were analyzed using an MGI metargetCOVID software version 2.0.1.230403 workflow, including data cleaning, sequence alignment, and full-genome assembly.

### 2.3. Phylogenetic and Evolution Analysis

The evaluation of the quality of whole genomes, the Nextstrain clade, and the Pango lineage assignment and variant calling was performed using Nextclade version 2.14.1 [18]. All of the in-house genomes passed through the quality check, and 6 genomes from GISAID failed. Finally, 299 SARS-CoV-2 whole genomes were subjected to further analysis using a bioinformatics toolkit named Augur version 2.1.13 [19]. Augur provides a series of modules for phylogenetic analysis consisting of a number of tools that allow the user to filter and align sequences, infer trees, and integrate the phylogenetic analysis with metadata. In detail, we aligned all of the sequences using Mafft (version 7.505) [20] and trimmed the data in order to mask out 5′ and 3′ UTRs. The resulting alignment was reconstructed as a maximum-likelihood phylogeny using IQ-Tree (version 2.0.3) [21]. This tree was further refined using Augur and TreeTime (version 0.9.2) [22] to add ancestral reconstructions and was visualized using the web tool Auspice.

## 3. Results

**Figure 1:**
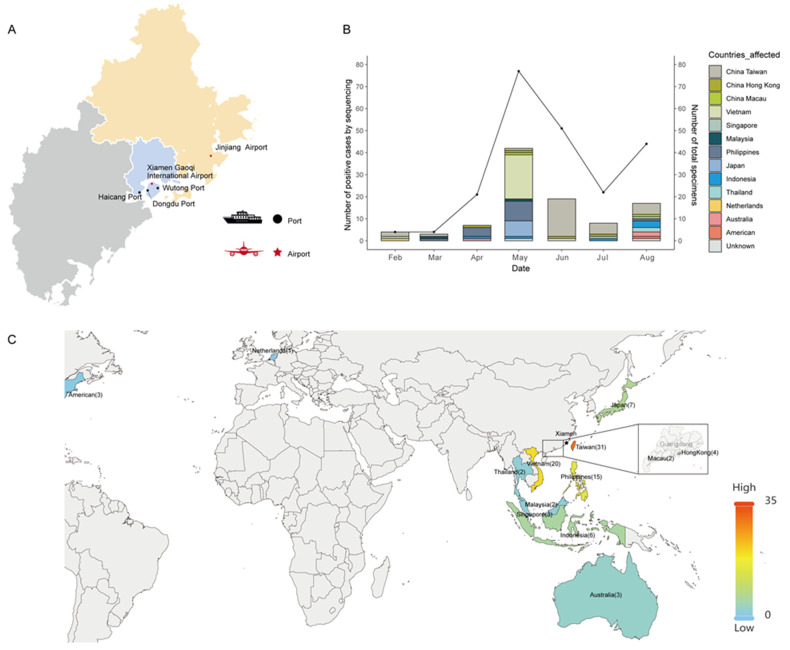
Spatial and temporal distribution of imported SARS-CoV-2 cases for countries or regions from which travelers entered Xiamen. (**A**) The location of the five international sampling sites for imported SARS-CoV-2 cases through Xiamen city. Black circles denote the ports accessed by ferry. The red stars correspond to the airports accessed by flight. (**B**) Monthly distribution of imported SARS-CoV-2 variants isolated and sequenced from incoming travelers at Xiamen International Airport and the Port of Xiamen entry border between February and August 2023. The bars represent the number of SARS-CoV-2 sequenced cases. The colors of the bars represent the countries or regions from which travelers entered Xiamen. The plotted line represents the number of sampling specimens. (**C**) Map showing the number of sequences with recorded travel history per country or region to describe geographic transmission lines to Xiamen, with more intense red colors representing more sequenced samples. Though few sequences are available from Hong Kong and Macau, these travel history data are highlighted to provide further details. The black stars correspond to the location of Xiamen city.

A total of 223 samples were collected from five sampling locations encompassing the main international airports and ports in Xiamen from February to August 2023, including Xiamen Gaoqi International Airport (105), Jinjiang Airport (29), Wutong Port (58), Dongdu Port (23), and Haicang Port (8). After the RT-qPCR assay, 100 high-quality genomic sequences from positive samples with cycle threshold (CT) values less than 32 were selected for next-generation sequencing, which accounted for 44.8% of the total cases (Figure 1A). The monthly positivity rates from February to August were 100% (4/4), 75% (3/4), 33.3% (7/21), 54.5% (42/77), 37.3% (19/51), 36.4% (8/22), and 38.6% (17/44), respectively. In addition, the samples from ports were mainly from Taiwan (18), Japan (7), and Vietnam (15), while the samples from airports were distributed in other countries or regions (Supplementary File Table S1). The relationship between the number of total imported cases and the number of sequences obtained, along with individual travel history data to Xiamen, is depicted in Figure 1B and Table 1. The number of imported cases remained relatively low from February to April but showed a significant increase starting from May 1, coinciding with the tourist season for Xiamen city. One instance of imported increasing cases was observed, consistent with the sequenced cases.

Significant efforts were made to obtain metadata for all cases, especially individual travel history data, as they may provide insights into the origins of variants introduced from neighboring countries or regions (Table 1). However, three cases collected from ports for describing geographic transmission were eliminated due to a lack of recorded travel history. Among the 97 cases with recorded travel history from the aforementioned entry points in Xiamen, the individuals came from 12 different countries and regions. Specifically, 31 cases were from Taiwan, while the others originated from Vietnam (20), the Philippines (15), Japan (7), Indonesia (5), Hong Kong (4), Singapore (3), Malaysia (3), Macau (2), Australia (3), Thailand (2), America (1), and the Netherlands (1). The transmission origins of these travel cases, for which genomic data were available, are illustrated in Figure 1C, with a particular focus on Hong Kong and Macau, regions that often go unnoticed on world maps. The availability of travel history data is therefore crucial in characterizing the viral population in these countries and regions, even in the absence of an abundance of samples or knowledge regarding the origin of local viruses circulating in Xiamen, a well-known tourist port city on the southeast coast of China.

**Figure 2:**
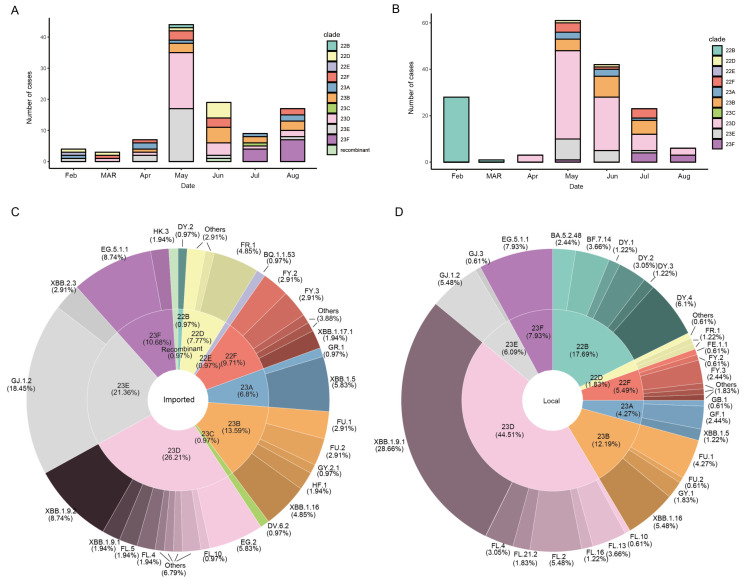
Clade statistics of imported SARS-CoV-2 variants in Xiamen over time. (**A**) Clade diversity of imported SARS-CoV-2 variants isolated and sequenced from incoming travelers at Xiamen International Airport and the Port of Xiamen entry border between February and August 2023. (**B**) Clade diversity of local cases isolated and sequenced in Xiamen and deposited from GISAID between February and August 2023. (**C**,**D**) The composition of imported (**C**) and local (**D**) cases for SARS-CoV-2 variants in Xiamen during the surveillance period. Among all of the local cases detected by Xiamen CDC in 2023, a total of 166 laboratory-confirmed COVID-19 cases were selected for genomic analysis based on high-quality sequencing data through the GISAID database in this study. The inner doughnut represents the composition of the clades. The outer doughnut represents the detailed Pango lineage within each clade.

The available genomes were analyzed using Nextclade software (version 2.14.1). As illustrated in Figure 2, a transition from clade 22 to clade 23 was observed starting from 2023. The XBB lineages and its sublineages accounted for approximately 89.7% of the sequenced samples. Within the new waves of the XBB subvariant, XBB.2.3 variants were first detected in imported cases in Xiamen on February 27 [23]. Subsequently, various sublineages of XBB dominated the imported cases from February 2023 onwards, and the distribution of lineages continued to evolve over time. In May 2023, clades 23E (GJ.1.2) and 23D (XBB.1.9.1 and XBB.1.9.2) rapidly became dominant in imported cases in Xiamen. Notably, clade 23E underwent a selective sweep and emerged as the prevailing clade, while clade 23D and the newly identified clade 23F also increased in prevalence during July and August (Figure 2A). In the meantime, SARS-CoV-2 genomic sequences detected by Xiamen CDC from February to August 2023 were downloaded from the GISAID database, regarding circulating variants isolated in Xiamen as “local” (See Table S2). Among all of the local cases downloaded, a total of 164 laboratory-confirmed COVID-19 cases were selected for genomic analysis based on high-quality sequencing data. Among these local infections, detailed analysis indicated their association with 8 clades, while the imported cases demonstrated diversity across 11 different clades (Figure 2C,D). Furthermore, prevailing clade 22B decreased in early 2023 following policy adjustments. Then, clade 23D first emerged in April 2023 and increased rapidly in May 2023, accounting for 44.5% as the dominant strain in local samples from Xiamen, while clades 22B (17.7%) and 23B (12.2%) took the second and third spots in terms of prevalence, respectively (Figure 2B,D). Moreover, clade 23F demonstrated a consistent upward trend in the incidence of both local and imported cases beginning in July. However, clades 23D and 23E accounted for 26.2% and 21.4% of the imported sequences, respectively, with relatively balanced proportions. Notably, the proportion of the 22B subvariant differed significantly between local (17.7%) and imported (1.0%) cases.

**Figure 3:**
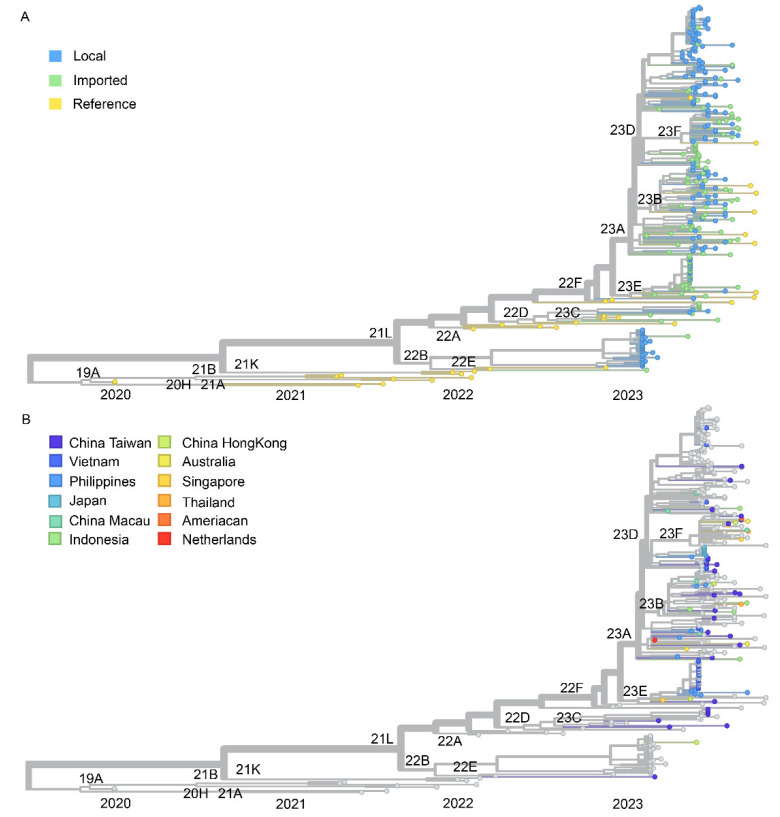
Phylogenetic tree of SARS-COV-2 genomic sequences for imported and local variants in Xiamen. Above is the phylogenetic tree of complete high-quality SARS-CoV-2 genomes generated in this study. The phylogenetic tree was visualized with tools provided by the Nextstrain server at “https://nextstrain.org/” (accessed on 4 December 2023). Tips with a circle indicate the genomic sequences, including imported, local, and reference viruses in this study. The color of the circle corresponds to the different clades in (**A**) and different origin types in (**B**).

In terms of Nextstrain clade analysis, the tree was constructed using 100 high-coverage genomic sequences of imported SARS-CoV-2 variants, 164 local sequences in Xiamen, and 35 references from GISAID (See Table S3). The estimated location-annotated phylogenies enabled us to track the geographic spread of SARS-CoV-2 over time for the XBB subvariant (Figure 3). Detailed analysis indicated that these local infections had a wider clade range compared with the imported cases, especially for the 22B and 23D clades. Regarding imported cases, the phylogenetic tree showed that most of the imported sequences belonged to genetic group 22F XBB. Indeed, a significant homology can be observed between the imported and local sequences within the 23 clades, as indicated by the phylogenetic tree analysis. This outcome strongly indicates a notable correlation between the source of local COVID-19 variants in Xiamen and the imported samples. In our analysis of imported sequences 23D and 23E, which dominated in early 2023, we inferred a minimum number of 47 introduction events into Xiamen, with a minimum of 7, 9, 18, and 7 events originating from Taiwan, the Philippines, Vietnam, and Japan (Table 1), respectively. We found that the introduction events of newly dominant clade 23F were evenly derived from the different countries or regions, such as Taiwan, Singapore, Hong Kong, Australia, Macau, and Thailand, which also suggested that clade 23F had been dominant worldwide at that same time. To summarize further, we can see that Taiwan, the Philippines, and Vietnam are the main source countries for imported cases. Among them, samples from Taiwan and the Philippines are composed of nine and six clades, respectively, while samples from Vietnam are only composed of three clades. This result suggests that the diversity of the imported SARS-CoV-2 variants in Xiamen mainly originates from Taiwan and the Philippines. Interestingly, it can be observed that in May all the Japanese samples belonged to the 23D clade, while the Vietnamese samples were predominantly from the 23E clade, suggesting that each country experienced different prevailing variants during that period. However, because of the differences in sequencing efforts and travel histories obtained from the imported cases, we cannot dismiss the possibility of instances of false origin in these cases. Nonetheless, all the spatial trajectory plots imply the presence of SARS-CoV-2 lineages circulating in Xiamen during the study period.

## 4. Discussion

In this study, we present findings on the trend of COVID-19 cases and the transmission patterns of imported SARS-CoV-2 variants among travelers in Xiamen, China from February 2023 to August 2023, as identified through border screening at Xiamen International Airport and the Port of Xiamen. The data revealed a major increase in imported cases in May 2023, with a peak of 42 sequences. Additionally, a smaller peak occurred in August, aligning with the global wave of COVID-19 caused by XBB subvariants in 2023 from “https://nextstrain.org/” (accessed on 4 December 2023). Epidemic investigations and phylogenetic analyses demonstrated that imported transmission was a common factor contributing to most of the diversities of SASR-CoV-2 variants in Xiamen during the study period. In general, our data showed continuing introduction with imported infections after December 2022, which highlights the change of the dynamic zero-COVID policy implemented in China, considering the high transmissibility of XBB subvariants, which had exhibited significant drift relative to BA.4 and BA.5 [8], the lineages on which the bivalent Pfizer–BioNTech vaccine was based [24].

In early 2023, BF.7, BQ.1.1, and BA.5.2 were the most prevalent subvariants in mainland China, including Beijing, Hebei province, Guangzhou municipality, and Shanghai municipality [25,26]. Simultaneously, the number of XBB mutant strains was rapidly increasing globally and competing with BQ.1 and BA.2.75, making it the dominant strain worldwide within a short time due to its enhanced fitness [7,27,28]. A recent report indicated the occurrence of two new waves of dominant infections driven by BA.5.2 and XBB.1 in Malaysia from June 2022 to April 2023, followed by an eighth wave led by XBB.1.9 [29]. This phenomenon aligns with genomic surveillance findings in Wisconsin, USA [30]. Moreover, the composition of XBB subvariants exhibited continuous variability worldwide [31,32]. As is known, even though there are more than 1.28 billion people vaccinated against COVID-19 in China, the updated vaccine against the XBB subvariants with immune-escaping mutations had not been approved and used in Xiamen during the study period. Thus, we speculate that due to the relatively recent policy adjustments, the population in mainland China still exhibits an immunological gap for the XBB lineages and its sublineages.

The predominant emerging variants observed in mainland China chiefly enter through imported cases from other countries [25,33,34]. Despite the dominance of the XBB subvariant, the composition of the remaining subvariants differs among countries and regions, warranting further investigation. It was observed that, although these local infections had a wider clade range compared with the imported cases, the imported samples showed greater clade diversity. We hypothesize that, due to the immunological gap in mainland China, whenever a new variant is introduced, it will first became prevalent in the local population and further undergo diversification, thereby showing broader clade differentiation locally in Xiamen, until it is replaced by new variants that have established a global trend, which is also consistent with the greater clade diversity for imported sequences. Consider the BA.5.2 variant as an example: its global proportion rapidly decreased from approximately 43% at the beginning of the year to less than 10% in early May 2023. It is speculated that the distribution of imported cases more accurately reflects the global strain distribution, while the local epidemic situation lags behind to some extent [35,36]. In fact, due to the vast territory of China and the significant climate differences between the north and south, the prevalence of BA.5.2 varies across different provinces in China [37]. This decline reflects both the local prevalence of the BA.5.2 variant during the study period in Xiamen and the lagging trend in local variant composition. Interestingly, the BA.5.2 variant only accounted for 0.97% of the imported sequences, suggesting that the predominant lineages in the travelers’ origin countries are more likely to dominate imported cases, thereby gaining an advantage in international transmission routes. Additionally, although the first XBB.2.3 variant detected in China originated from imported samples in Xiamen, and the 23E variants accounted for a certain proportion of the imported samples at the Port of Xiamen, this variant did not effectively spread locally in Xiamen, aligning with the global trend of XBB.2.3. Overall, XBB.1.5 replaced the previously dominant Omicron variants globally and co-circulated with other XBB subvariants (XBB.1.9, XBB.1.16, and XBB.2.3). By October 2023, the EG.5.1 subvariant had rapidly spread and become the prevailing strain worldwide since its initial detection in early 2023 from “https://nextstrain.org/” (accessed on 4 December 2023) [38], aligning with the dynamic distribution of variants in imported cases in Xiamen. Notably, we observed that the dominant circulating EG.5 variant worldwide and in mainland China was also monitored in a timely manner and detected in the imported sequences. Overall, the imported genomic sequences do not represent the global situation of SARS-CoV-2 due to limitations regarding the origins of travelers and the sample numbers. Nevertheless, the identification of variants in imported cases with transmission advantages or immune evasion characteristics in imported cases offers critical support for the enhancement of COVID-19 surveillance and early warning systems.

In this study, we present the ongoing genomic sequencing efforts conducted in Xiamen, a renowned tourist port city on the southeast coast of China. These efforts involved meticulous collection of travel history metadata associated with incoming travelers at the Xiamen International Airport and the Port of Xiamen. Notably, we were able to include traveler cases from 12 different countries and regions, primarily located in Southeast Asia, with a focus on Taiwan, Vietnam, and the Philippines. Travelers from Taiwan exhibited the greatest diversity in variants with abundant cases, and a similar pattern was observed in the Philippines. This may be attributed to the close geographical proximity and increased exchanges between these regions. Taiwanese inputs accounted for the highest number of sequenced samples, which were evenly distributed across time and variant types, thus representing a significant portion. However, if a dominant strain was predominantly circulated in the nearby countries and areas abroad, the cases introduced into Xiamen would be the prevalent variant, as was the case with Japan and Vietnam. Additionally, although there were fewer imported sequences from countries such as Singapore, the United States, Australia, Japan, and the Netherlands, it is still evident that the detected foreign variant aligns closely with international trends at different time points. Notably, the XBB.2.3 variant strain identified in Singapore represents the first reported case of the XBB.2.3 sequence detected in our country.

Our findings indicate a significant contribution of neighboring countries to the spread of various SARS-CoV-2 lineages in Xiamen. During the study period, we observed a co-circulation peak of the 23E and 23D subvariants in the imported sequences to Xiamen, alongside a surge in the EG.5 subvariant. Significantly, the evolutionary trajectory of SARS-CoV-2 has shown continual progression, with a recent global shift observed from XBB to BA.2.86, exemplified by the emergence of the predominant JN.1 subvariant [39]. This surveillance study conducted at Xiamen International Airport and the Port of Xiamen provides a glimpse of China’s COVID-19 landscape and serves as an important tool for surveillance and early warning systems, considering both the frequent population exchange and the presence of highly transmissible circulating SARS-CoV-2 lineages.

## Figures and Tables

**Table 1:** SARS-CoV-2 clades identified from sequences imported to Xiamen at airports and ports from February to August 2023.

Country or Region Source	Clade (Numbers)	Date (Numbers)
America	23D (1)	August
Australia	23A (2)	April/August
	23F (1)	August
China—Hong Kong	23E (1)	April
	22B (1)	May
	23B (1)	June
	23F (1)	July
China—Macau	23A (1)	May
	23F (1)	August
China—Taiwan	22D (7)	February/March/June (5)
	22E (1)	February
	22F (3)	June (3)
	23A (2)	July/August
	23B (6)	June (3)/July/August (2)
	23C (1)	July
	23D (6)	May/ June (4)/July
	23E (1)	June
	23F (3)	July/August (2)
	Recombinant	June
Indonesia	22F (1)	August
	23B (3)	April/July/August
	23D (1)	August
Japan	23D (7)	May (7)
Malaysia	23D (2)	March/May
	23B (1)	May
The Netherlands	23A (1)	February
The Philippines	22F (3)	March/April /May
	23A (1)	April
	23B (2)	May (2)
	23D (3)	April /May (2)
	23E (6)	April/May (4)/August
Singapore	23E (1)	February
	23F (2)	July/August
Thailand	22F (1)	August
	23F (1)	August
Vietnam	22F (2)	May (2)
	23D (5)	May (5)
	23E (13)	May (13)
Unknown	22D (1)	May
	23B (1)	June
	23F (1)	August

## Data Availability

The data presented in this study will be available on request from the corresponding author.

## Supplementary Materials

The following supporting information can be downloaded at: https://www.mdpi.com/article/10.3390/v16010132/s1. Table S1: The details for all samples collected in this study at Xiamen airport and port.; Table S2: SARS-CoV-2 clades and lineages downloaded from GISAID at Xiamen; Table S3: SARS-CoV-2 reference sequences downloaded from GISAID.
