# Supplementary material for: Systematic Genomic Surveillance of SARS-CoV-2 at Xiamen International Airport and the Port of Xiamen Reveals the Importance of Incoming Travelers in Lineage Diversity
Source: Viruses. 2024 Jan 17;16(1):132. doi: 10.3390/v16010132 (PMC10821529; doi:10.3390/v16010132)
Supplement: Supplementary file 1 [file viruses-16-00132-s001.zip › viruses-2800821-supplementary.pdf]

Systematic Genomic Surveillance of SARS-CoV-2 at Xiamen International Airport and the Port of Xiamen Reveals the Importance of Incoming Travelers in Lineage Diversity

Ruiluan You [1,†], Ruotong Wu [2,†], Xijing Wang [2], Rao Fu [2,3], Ningshao Xia [2,3], Yixin Chen [2,3], Kunyu Yang [1,*] and Junyu Chen [2,3,*]

[1] Xiamen International Travel Healthcare Center, Xiamen Entry-Exit Inspection and Quarantine Bureau, Xiamen 361001, China;

[2] State Key Laboratory of Vaccines for Infectious Diseases, Xiang An Biomedicine Laboratory, Department of Laboratory Medicine, School of Public Health & School of Life Sciences, Xiamen University, Xiamen 361102, China;

[3] National Institute of Diagnostics and Vaccine Development in Infectious Diseases, State Key Laboratory of Molecular Vaccinology and Molecular Diagnostics, Collaborative Innovation Center of Biologic Products, National Innovation Platform for Industry-Education Integration in Vaccine Research, Xiamen University, Xiamen 361102, China

* Correspondence: yangkunyu@xmu.edu.cn (K.Y.); junyuchen@xmu.edu.cn (J.C.)

† These authors contributed equally to this work.

Table.1 The details for all samples collected in this study at Xiamen airport and port.

date	Gender	Sampling Location	Origin	CT_value	sequence
20230418		Dongdu Port	Unknown	34.56	No
20230513	Male	Dongdu Port	Vietnam	27.07	Yes
20230513	Male	Dongdu Port	Vietnam	24.78	Yes
20230513	Male	Dongdu Port	Vietnam	22.14	Yes
20230513	Male	Dongdu Port	Vietnam	19.33	Yes
20230513	Male	Dongdu Port	Vietnam	24.56	Yes
20230513	Male	Dongdu Port	Vietnam	26.21	Yes
20230513	Male	Dongdu Port	Vietnam	21.33	Yes
20230513	Male	Dongdu Port	Vietnam	23.09	Yes
20230513	Male	Dongdu Port	Vietnam	21.99	Yes
20230513	Male	Dongdu Port	Vietnam	33.39	No
20230513	Male	Dongdu Port	Vietnam	26.96	Yes
20230513	Male	Dongdu Port	Vietnam	25.28	Yes
20230513	Male	Dongdu Port	Vietnam	17.83	Yes
20230513	Male	Dongdu Port	Vietnam	23.37	Yes
20230513	Male	Dongdu Port	Vietnam	27.03	Yes
20230513	Male	Dongdu Port	Vietnam	22.19	Yes
20230602	Male	Dongdu Port	China Taiwan	21.34	Yes
20230602	Male	Dongdu Port	China Taiwan	22.91	Yes
20230602	Male	Dongdu Port	China Taiwan	14.65	Yes
20230602	Male	Dongdu Port	China Taiwan	20.69	Yes
20230602	Male	Dongdu Port	China Taiwan	31.22	Yes
20230602	Male	Dongdu Port	China Taiwan	32.22	No
20230525	Male	Haicang Port	Japan	31.4	Yes
20230525	Male	Haicang Port	Japan	23.74	Yes
20230525	Male	Haicang Port	Japan	28.16	Yes
20230525	Male	Haicang Port	Japan	24.46	Yes
20230525	Male	Haicang Port	Japan	28.85	Yes
20230525	Male	Haicang Port	Japan	32.21	Yes
20230525	Male	Haicang Port	Japan	19.23	Yes
20230611	Male	Haicang Port	Unknown	22.91	Yes
20230313		Jinjiang Airport	China Taiwan	24.52	Yes
20230330	Male	Jinjiang Airport	Philippines	22.05	Yes
20230428	Female	Jinjiang Airport	Philippines	30.52	Yes
20230428	Male	Jinjiang Airport	Philippines	31.45	Yes
20230429	Female	Jinjiang Airport	Philippines	27.03	Yes
20230506		Jinjiang Airport	Philippines	25.93	Yes
20230506		Jinjiang Airport	Philippines	29.21	Yes
20230509	Male	Jinjiang Airport	China HongKong	23.11	Yes
20230509	Female	Jinjiang Airport	Philippines	29.67	Yes

20230514	Male	Jinjiang Airport	Philippines	30.54	Yes
20230514	Male	Jinjiang Airport	China Macau	30.67	Yes
20230518	Male	Jinjiang Airport	Philippines	28.49	Yes
20230518	Female	Jinjiang Airport	Philippines	26.54	Yes
20230528	Male	Jinjiang Airport	Philippines	29.93	Yes
20230529	Male	Jinjiang Airport	Philippines	30.53	Yes
20230530	Male	Jinjiang Airport	Vietnam	25.84	Yes
20230530	Male	Jinjiang Airport	Philippines	27.84	Yes
20230530	Male	Jinjiang Airport	Vietnam	28.48	Yes
20230530	Male	Jinjiang Airport	Vietnam	30.66	Yes
20230530	Male	Jinjiang Airport	Vietnam	20.14	Yes
20230530	Male	Jinjiang Airport	China Taiwan	20.25	Yes
20230530	Male	Jinjiang Airport	Vietnam	32.12	Yes
20230602	Male	Jinjiang Airport	China Taiwan	29.79	Yes
20230602	Male	Jinjiang Airport	China Taiwan	28.82	Yes
20230611	Male	Jinjiang Airport	China Taiwan	18.3	Yes
20230619	Female	Jinjiang Airport	China HongKong	22.49	Yes
20230621	Female	Jinjiang Airport	China Taiwan	25.55	Yes
20230730	Male	Jinjiang Airport	China HongKong	25.45	Yes
20230811	Male	Jinjiang Airport	China Taiwan	25.81	Yes
20230421		Wutong Port	China Taiwan	32.07	No
20230511	Male	Wutong Port	China Taiwan	35.5	No
20230511	Female	Wutong Port	China Taiwan	34.37	No
20230513	Female	Wutong Port	China Taiwan	34.5	No
20230516	Male	Wutong Port	China Taiwan	34.32	No
20230516	Female	Wutong Port	China Taiwan	35.55	No
20230517	Male	Wutong Port	China Taiwan	32.7	No
20230518	Male	Wutong Port	China Taiwan	29.98	Yes
20230520	Female	Wutong Port	China Taiwan	32.04	No
20230520	Male	Wutong Port	China Taiwan	32.85	No
20230520	Female	Wutong Port	China Taiwan	33.33	No
20230523	Male	Wutong Port	China Taiwan	33.17	No
20230524	Male	Wutong Port	China Taiwan	32.97	No
20230530	Male	Wutong Port	China Taiwan	32.63	No
20230602	Male	Wutong Port	China Taiwan	25.19	Yes
20230604	Female	Wutong Port	China Taiwan	32.11	No
20230608	Male	Wutong Port	China Taiwan	32.07	No
20230609	Male	Wutong Port	China Taiwan	32.84	No
20230610	Female	Wutong Port	China Taiwan	31.31	No
20230610	Female	Wutong Port	China Taiwan	29.83	No
20230611	Female	Wutong Port	China Taiwan	31.31	Yes
20230611	Female	Wutong Port	China Taiwan	29.83	Yes
20230612	Female	Wutong Port	China Taiwan		No

20230613	Male	Wutong Port	China Taiwan	27.04	Yes
20230613	Female	Wutong Port	China Taiwan	28.77	Yes
20230615	Male	Wutong Port	China Taiwan	32.96	No
20230615	Male	Wutong Port	China Taiwan	32.41	No
20230616	Male	Wutong Port	China Taiwan	25.39	Yes
20230616	Male	Wutong Port	China Taiwan	24.06	Yes
20230617	Male	Wutong Port	China Taiwan	27	Yes
20230617	Male	Wutong Port	China Taiwan	32.6	No
20230618	Female	Wutong Port	China Taiwan	33.63	No
20230620	Female	Wutong Port	China Taiwan	33.32	No
20230624	Male	Wutong Port	China Taiwan	32.32	No
20230630	Male	Wutong Port	China Taiwan	32.53	No
20230701	Female	Wutong Port	China Taiwan	34.24	No
20230704	Female	Wutong Port	China Taiwan	32.31	No
20230705	Male	Wutong Port	China Taiwan	34	No
20230706	Female	Wutong Port	China Taiwan	33.49	No
20230706	Male	Wutong Port	China Taiwan	32.33	No
20230709	Male	Wutong Port	China Taiwan	32.53	No
20230712	Male	Wutong Port	China Taiwan	24.16	Yes
20230719	Male	Wutong Port	China Taiwan	26.58	Yes
20230719	Male	Wutong Port	China Taiwan	32.44	No
20230721	Male	Wutong Port	China Taiwan	33.2	No
20230801	Male	Wutong Port	China Taiwan	33.37	No
20230801	Male	Wutong Port	China Taiwan	34.66	No
20230801	Male	Wutong Port	Unknown	33.91	No
20230803	Male	Wutong Port	China Taiwan	33.36	No
20230804	Male	Wutong Port	China Taiwan	32.73	No
20230808	Female	Wutong Port	China Taiwan	31.61	Yes
20230809	Female	Wutong Port	China Taiwan	33.48	No
20230811	Male	Wutong Port	China Taiwan	26.92	Yes
20230811	Male	Wutong Port	China Taiwan	28.54	Yes
20230812	Male	Wutong Port	Unknown	28.04	Yes
20230814	Male	Wutong Port	China Taiwan	32.66	No
20230819	Male	Wutong Port	China Taiwan	35.69	No
20230821	Male	Wutong Port	China Taiwan	33.01	No
20230210	Female	Xiamen Gaoqi international Airport	China Taiwan	27.98	Yes
20230210	Male	Xiamen Gaoqi international Airport	Netherlands	29.7	Yes
20230218	Female	Xiamen Gaoqi international Airport	China Taiwan	27	Yes
20230227	Male	Xiamen Gaoqi international Airport	Singapore	23.65	Yes
20230309	Female	Xiamen Gaoqi international Airport	Malaysia	21.81	Yes
20230317		Xiamen Gaoqi international Airport	American	32.67	No
20230403		Xiamen Gaoqi international Airport	Philippines	24.66	Yes
20230405		Xiamen Gaoqi international Airport	Korea	32.33	No

20230410		Xiamen Gaoqi international Airport	China Taiwan	32.86	No
20230413		Xiamen Gaoqi international Airport	China Taiwan	32.37	No
20230415		Xiamen Gaoqi international Airport	American	32.15	No
20230418		Xiamen Gaoqi international Airport	Australia	23.93	Yes
20230419		Xiamen Gaoqi international Airport	Unknown	34.86	No
20230420		Xiamen Gaoqi international Airport	Malaysia	34.83	No
20230421		Xiamen Gaoqi international Airport	Indonesia	34.51	No
20230422		Xiamen Gaoqi international Airport	Unknown	32.17	No
20230425		Xiamen Gaoqi international Airport	Indonesia	27.21	Yes
20230425		Xiamen Gaoqi international Airport	Singapore	34.54	No
20230426		Xiamen Gaoqi international Airport	China HongKong	26.74	Yes
20230426		Xiamen Gaoqi international Airport	Canada	32	No
20230427		Xiamen Gaoqi international Airport	Indonesia	33.59	No
20230428		Xiamen Gaoqi international Airport	China Taiwan	34.6	No
20230503		Xiamen Gaoqi international Airport	Australia	32.38	No
20230504		Xiamen Gaoqi international Airport	Singapore	32.68	No
20230505		Xiamen Gaoqi international Airport	Unknown	33.49	No
20230505		Xiamen Gaoqi international Airport	Unknown	32.88	No
20230509	Male	Xiamen Gaoqi international Airport	Malaysia	27.65	Yes
20230509	Male	Xiamen Gaoqi international Airport	Vietnam	32.21	No
20230509	Female	Xiamen Gaoqi international Airport	Malaysia	28.27	Yes
20230510	Male	Xiamen Gaoqi international Airport	Australia	32.69	No
20230510	Female	Xiamen Gaoqi international Airport	Malaysia	32.82	No
20230511	Female	Xiamen Gaoqi international Airport	Vietnam	35.3	No
20230512	Male	Xiamen Gaoqi international Airport	Malaysia	33.16	No
20230513	Male	Xiamen Gaoqi international Airport	Singapore	33.13	No
20230516	Female	Xiamen Gaoqi international Airport	China Taiwan	34.2	No
20230517	Male	Xiamen Gaoqi international Airport	Singapore	34.67	No
20230519	Female	Xiamen Gaoqi international Airport	China Macau	34.06	No
20230524	Female	Xiamen Gaoqi international Airport	Thailand	35.44	No
20230524	Female	Xiamen Gaoqi international Airport	Singapore	32.37	No
20230524	Male	Xiamen Gaoqi international Airport	China Taiwan	32.19	No
20230525	Male	Xiamen Gaoqi international Airport	China Taiwan	32.86	No
20230526	Male	Xiamen Gaoqi international Airport	China Taiwan	33.97	No
20230528	Male	Xiamen Gaoqi international Airport	China Taiwan	33.11	No
20230528	Female	Xiamen Gaoqi international Airport	Singapore	32.53	No
20230529	Female	Xiamen Gaoqi international Airport	Vietnam	24.93	No
20230529	Female	Xiamen Gaoqi international Airport	China Taiwan	34.45	No
20230603		Xiamen Gaoqi international Airport	China Taiwan	33.9	No
20230605	Male	Xiamen Gaoqi international Airport	China Taiwan	32.15	No
20230607	Male	Xiamen Gaoqi international Airport	Unknown	34.01	No
20230611	Male	Xiamen Gaoqi international Airport	Indonesia	34.48	No
20230612	Male	Xiamen Gaoqi international Airport	China Taiwan		No

20230613	Male	Xiamen Gaoqi international Airport	Unknown	33.21	No
20230615	Female	Xiamen Gaoqi international Airport	Thailand	33.29	No
20230615		Xiamen Gaoqi international Airport	Indonesia	32.33	No
20230618	Female	Xiamen Gaoqi international Airport	Thailand	32.46	No
20230618	Male	Xiamen Gaoqi international Airport	China Taiwan	34.5	No
20230619	Female	Xiamen Gaoqi international Airport	Philippines	32.65	No
20230622	Female	Xiamen Gaoqi international Airport	Philippines	32.12	No
20230622	Male	Xiamen Gaoqi international Airport	Vietnam	32.7	No
20230623	Male	Xiamen Gaoqi international Airport	Thailand	32.47	No
20230626	Male	Xiamen Gaoqi international Airport	Australia	33.59	No
20230626	Female	Xiamen Gaoqi international Airport	China Taiwan	32.4	No
20230630	Male	Xiamen Gaoqi international Airport	Thailand	32.65	No
20230703	Female	Xiamen Gaoqi international Airport	Singapore	33.14	No
20230707	Male	Xiamen Gaoqi international Airport	China Taiwan	33.18	No
20230708	Female	Xiamen Gaoqi international Airport	Singapore	32.28	No
20230708	Male	Xiamen Gaoqi international Airport	Unknown	33.57	No
20230711	Female	Xiamen Gaoqi international Airport	Australia	32.05	No
20230715	Male	Xiamen Gaoqi international Airport	Vietnam	33.24	No
20230715	Male	Xiamen Gaoqi international Airport	Singapore	26.13	Yes
20230715	Male	Xiamen Gaoqi international Airport	China Taiwan	27.26	Yes
20230719	Male	Xiamen Gaoqi international Airport	China Taiwan	22.45	Yes
20230721	Female	Xiamen Gaoqi international Airport	Singapore	34.59	No
20230727	Male	Xiamen Gaoqi international Airport	Indonesia	23.55	Yes
20230729	Male	Xiamen Gaoqi international Airport	China Taiwan	26.94	Yes
20230806	Female	Xiamen Gaoqi international Airport	Singapore	32.96	No
20230808	Male	Xiamen Gaoqi international Airport	Singapore	33.24	No
20230808	Male	Xiamen Gaoqi international Airport	Indonesia	25.35	Yes
20230808	Female	Xiamen Gaoqi international Airport	Indonesia	31.91	Yes
20230808	Male	Xiamen Gaoqi international Airport	China Taiwan	27.11	Yes
20230811	Female	Xiamen Gaoqi international Airport	Singapore	24.07	Yes
20230812	Female	Xiamen Gaoqi international Airport	Thailand	20.1	Yes
20230812	Male	Xiamen Gaoqi international Airport	American	27.55	Yes
20230814	Female	Xiamen Gaoqi international Airport	Australia	33.2	No
20230814	Female	Xiamen Gaoqi international Airport	Singapore	34.65	No
20230815	Male	Xiamen Gaoqi international Airport	Vietnam	34.37	No
20230815	Male	Xiamen Gaoqi international Airport	Australia	32.9	No
20230815	Male	Xiamen Gaoqi international Airport	Japan	35.34	No
20230815	Female	Xiamen Gaoqi international Airport	Japan	35.48	No
20230816	Male	Xiamen Gaoqi international Airport	Singapore	32.23	No
20230817	Female	Xiamen Gaoqi international Airport	Australia	32.6	No
20230818	Female	Xiamen Gaoqi international Airport	Singapore	32.37	No
20230821	Female	Xiamen Gaoqi international Airport	Indonesia	32.76	No
20230822	Male	Xiamen Gaoqi international Airport	Japan	34.21	No

20230823	Female	Xiamen Gaoqi international Airport	China Macau	18.8	Yes
20230823	Female	Xiamen Gaoqi international Airport	Indonesia	24.93	Yes
20230824	Female	Xiamen Gaoqi international Airport	Vietnam	34.44	No
20230824	Female	Xiamen Gaoqi international Airport	Australia	21.54	Yes
20230824	Male	Xiamen Gaoqi international Airport	Philippines	21.08	Yes
20230825	Male	Xiamen Gaoqi international Airport	Australia	21.21	Yes
20230826	Female	Xiamen Gaoqi international Airport	Thailand	31.37	Yes
20230828	Female	Xiamen Gaoqi international Airport	Singapore	32.82	No
20230830	Female	Xiamen Gaoqi international Airport	Indonesia	33.52	No
20230831	Female	Xiamen Gaoqi international Airport	Singapore	32.98	No
20230831	Female	Xiamen Gaoqi international Airport	Philippines	32.24	No

Table.2 SARS-CoV-2 clades and lineages downloaded from GSAID at Xiamen

name	date	clade	Nextclade_pango	partiallyAliased	clade_who	clade_display	origin	gisaid_epi_isl
hCoV-19/Fujian/FCDC-2023010676/2023	2023/2/1	22B	DY.4	BA.5.2.48.4	Omicron	22B (BA.5)	GISAID	EPI_ISL_16977173
hCoV-19/Fujian/FCDC-2023010677/2023	2023/2/1	22B	BF.7.14	BA.5.2.1.7.14	Omicron	22B (BA.5)	GISAID	EPI_ISL_16977174
hCoV-19/Fujian/FCDC-2023010678/2023	2023/2/1	22B	DY.4	BA.5.2.48.4	Omicron	22B (BA.5)	GISAID	EPI_ISL_16977175
hCoV-19/Fujian/FCDC-2023010679/2023	2023/2/1	22B	DY.2	BA.5.2.48.2	Omicron	22B (BA.5)	GISAID	EPI_ISL_16977176
hCoV-19/Fujian/FCDC-2023010680/2023	2023/2/1	22B	DY.2	BA.5.2.48.2	Omicron	22B (BA.5)	GISAID	EPI_ISL_16977177
hCoV-19/Fujian/FCDC-	2023/2/1	22B	BF.7.14	BA.5.2.1.7.14	Omicron	22B (BA.5)	GISAID	EPI_ISL_16977178

2023010681/2023								
hCoV-19/Fujian/FCDC-2023010682/2023	2023/2/1	22B	DY.4	BA.5.2.48.4	Omicron	22B (BA.5)	GISAID	EPI_ISL_16977179
hCoV-19/Fujian/FCDC-2023010683/2023	2023/2/1	22B	DY.1	BA.5.2.48.1	Omicron	22B (BA.5)	GISAID	EPI_ISL_16977180
hCoV-19/Fujian/FCDC-2023010684/2023	2023/2/1	22B	DY.2	BA.5.2.48.2	Omicron	22B (BA.5)	GISAID	EPI_ISL_16977181
hCoV-19/Fujian/FCDC-2023010685/2023	2023/2/1	22B	DY.2	BA.5.2.48.2	Omicron	22B (BA.5)	GISAID	EPI_ISL_16977182
hCoV-19/Fujian/FCDC-2023020801/2023	2023/2/1	22B	BF.7.14.1	BA.5.2.1.7.14.1	Omicron	22B (BA.5)	GISAID	EPI_ISL_16977215
hCoV-19/Fujian/FCDC-2023020802/2023	2023/2/1	22B	DY.4	BA.5.2.48.4	Omicron	22B (BA.5)	GISAID	EPI_ISL_16977216
hCoV-19/Fujian/FCDC-2023020804/2023	2023/2/1	22B	BA.5.2.48	BA.5.2.48	Omicron	22B (BA.5)	GISAID	EPI_ISL_16977217
hCoV-19/Fujian/FCDC-2023020805/2023	2023/2/1	22B	BA.5.2.48	BA.5.2.48	Omicron	22B (BA.5)	GISAID	EPI_ISL_16977218
hCoV-19/Fujian/FC	2023/2/1	22B	BF.7.14	BA.5.2.1.7.14	Omicron	22B (BA.5)	GISAID	EPI_ISL_16977219

DC-2023020806/2023								
hCoV-19/Fujian/FCDC-2023020807/2023	2023/2/1	22B	DY.4	BA.5.2.48.4	Omicron	22B (BA.5)	GISAID	EPI_ISL_16977220
hCoV-19/Fujian/FCDC-2023020808/2023	2023/2/1	22B	DY.2	BA.5.2.48.2	Omicron	22B (BA.5)	GISAID	EPI_ISL_16977221
hCoV-19/Fujian/FCDC-2023020809/2023	2023/2/1	22B	DY.4	BA.5.2.48.4	Omicron	22B (BA.5)	GISAID	EPI_ISL_16977222
hCoV-19/Fujian/FCDC-020811/2023	2023/2/1	22B	BF.7.14.1	BA.5.2.1.7.14.1	Omicron	22B (BA.5)	GISAID	EPI_ISL_16977223
hCoV-19/Fujian/FCDC-2023020812/2023	2023/2/1	22B	DY.4	BA.5.2.48.4	Omicron	22B (BA.5)	GISAID	EPI_ISL_16977224
hCoV-19/Fujian/FCDC-2023020813/2023	2023/2/1	22B	BA.5.2.48	BA.5.2.48	Omicron	22B (BA.5)	GISAID	EPI_ISL_16977225
hCoV-19/Fujian/FCDC-2023020814/2023	2023/2/1	22B	DY.4	BA.5.2.48.4	Omicron	22B (BA.5)	GISAID	EPI_ISL_16977226
hCoV-19/Fujian/FCDC-021502/2023	2023/2/7	22B	BF.7.14	BA.5.2.1.7.14	Omicron	22B (BA.5)	GISAID	EPI_ISL_17072222
hCoV-19/Fujian/FC	2023/2/7	22B	DY.3	BA.5.2.48.3	Omicron	22B (BA.5)	GISAID	EPI_ISL_17072223

DC-021504/2023								
hCoV-19/Fujian/FCDC-021505/2023	2023/2/7	22B	BA.5.2.48	BA.5.2.48	Omicron	22B (BA.5)	GISAID	EPI_ISL_17072224
hCoV-19/Fujian/FCDC-021506/2023	2023/2/7	22B	DY.4	BA.5.2.48.4	Omicron	22B (BA.5)	GISAID	EPI_ISL_17072225
hCoV-19/Fujian/FCDC-030102/2023	2023/2/17	22B	DY.3	BA.5.2.48.3	Omicron	22B (BA.5)	GISAID	EPI_ISL_17100857
hCoV-19/Fujian/FCDC-030101/2023	2023/2/23	22B	DY.4	BA.5.2.48.4	Omicron	22B (BA.5)	GISAID	EPI_ISL_17100858
hCoV-19/Fujian/FCDC-2023030601/2023	2023/3/5	22B	DY.1	BA.5.2.48.1	Omicron	22B (BA.5)	GISAID	EPI_ISL_17261995
hCoV-19/Fujian/FCDC-XM042801/2023	2023/4/27	23D	XBB.1.9.1	XBB.1.9.1	Omicron	23D (XBB.1.9)	GISAID	EPI_ISL_17689440
hCoV-19/Fujian/FCDC-XM042803/2023	2023/4/27	23D	XBB.1.9.1	XBB.1.9.1	Omicron	23D (XBB.1.9)	GISAID	EPI_ISL_17689441
hCoV-19/Fujian/FCDC-XM042802/2023	2023/4/27	23D	XBB.1.9.1	XBB.1.9.1	Omicron	23D (XBB.1.9)	GISAID	EPI_ISL_17689442
hCoV-19/Fujian/FCDC-XM050608/2023	2023/5/4	23D	XBB.1.9.1	XBB.1.9.1	Omicron	23D (XBB.1.9)	GISAID	EPI_ISL_17689443

hCoV-19/Fujian/FCDC-XM050604/2023	2023/5/5	23D	XBB.1.9.1	XBB.1.9.1	Omicron	23D (XBB.1.9)	GISAID	EPI_ISL_17689444
hCoV-19/Fujian/FCDC-XM051519/2023	2023/5/13	23D	XBB.1.9.1	XBB.1.9.1	Omicron	23D (XBB.1.9)	GISAID	EPI_ISL_17704036
hCoV-19/Fujian/FCDC-XM051518/2023	2023/5/13	23D	XBB.1.9.1	XBB.1.9.1	Omicron	23D (XBB.1.9)	GISAID	EPI_ISL_17704037
hCoV-19/Fujian/FCDC-XM051517/2023	2023/5/13	22D	BN.1.2	BA.2.75.5.1.2	Omicron	22D (BA.2.75)	GISAID	EPI_ISL_17704038
hCoV-19/Fujian/FCDC-XM051516/2023	2023/5/13	23D	XBB.1.9.1	XBB.1.9.1	Omicron	23D (XBB.1.9)	GISAID	EPI_ISL_17704039
hCoV-19/Fujian/FCDC-XM051523/2023	2023/5/14	23E	GJ.1.2	XBB.2.3.3.1.2	Omicron	23E (XBB.2.3)	GISAID	EPI_ISL_17704040
hCoV-19/Fujian/FCDC-XM051527/2023	2023/5/14	22F	FY.2.1	XBB.1.22.1.2.1	Omicron	22F (XBB)	GISAID	EPI_ISL_17704041
hCoV-19/Fujian/FCDC-XM051529/2023	2023/5/14	23E	GJ.1.2	XBB.2.3.3.1.2	Omicron	23E (XBB.2.3)	GISAID	EPI_ISL_17704042
hCoV-19/Fujian/FCDC-	2023/5/14	23E	GJ.1.2	XBB.2.3.3.1.2	Omicron	23E (XBB.2.3)	GISAID	EPI_ISL_17704043

XM051520/2023								
hCoV-19/Fujian/FCDC-XM051525/2023	2023/5/14	23E	GJ.1.2	XBB.2.3.3.1.2	Omicron	23E (XBB.2.3)	GISAID	EPI_ISL_17704044
hCoV-19/Fujian/FCDC-XM051522/2023	2023/5/14	23E	GJ.1.2	XBB.2.3.3.1.2	Omicron	23E (XBB.2.3)	GISAID	EPI_ISL_17704045
hCoV-19/Fujian/FCDC-XM051528/2023	2023/5/14	23E	GJ.1.2	XBB.2.3.3.1.2	Omicron	23E (XBB.2.3)	GISAID	EPI_ISL_17704046
hCoV-19/Fujian/FCDC-XM051524/2023	2023/5/14	23E	GJ.1.2	XBB.2.3.3.1.2	Omicron	23E (XBB.2.3)	GISAID	EPI_ISL_17704047
hCoV-19/Fujian/FCDC-XM051530/2023	2023/5/14	23E	GJ.1.2	XBB.2.3.3.1.2	Omicron	23E (XBB.2.3)	GISAID	EPI_ISL_17704048
hCoV-19/Fujian/FCDC-XM051521/2023	2023/5/14	23E	GJ.1.2	XBB.2.3.3.1.2	Omicron	23E (XBB.2.3)	GISAID	EPI_ISL_17704049
hCoV-19/Fujian/FCDC-XM051602/2023	2023/5/20	23D	XBB.1.9.1	XBB.1.9.1	Omicron	23D (XBB.1.9)	GISAID	EPI_ISL_17729845
hCoV-19/Fujian/FCDC-XM051603/2023	2023/5/20	23D	XBB.1.9.1	XBB.1.9.1	Omicron	23D (XBB.1.9)	GISAID	EPI_ISL_17729846
hCoV-19/Fujian/FC	2023/5/20	23D	XBB.1.9.1	XBB.1.9.1	Omicron	23D (XBB.1.9)	GISAID	EPI_ISL_17729847

DC-XM051604/2023								
hCoV-19/Fujian/FCDC-XM051605/2023	2023/5/20	23D	XBB.1.9.1	XBB.1.9.1	Omicron	23D (XBB.1.9)	GISAID	EPI_ISL_17729849
hCoV-19/Fujian/FCDC-XM051606/2023	2023/5/20	23D	XBB.1.9.1	XBB.1.9.1	Omicron	23D (XBB.1.9)	GISAID	EPI_ISL_17729851
hCoV-19/Fujian/FCDC-XM051607/2023	2023/5/20	22D	FR.1.1	BA.2.75.5.1.2.3.1.1	Omicron	22D (BA.2.75)	GISAID	EPI_ISL_17729852
hCoV-19/Fujian/FCDC-XM051608/2023	2023/5/20	23D	XBB.1.9.1	XBB.1.9.1	Omicron	23D (XBB.1.9)	GISAID	EPI_ISL_17729853
hCoV-19/Fujian/FCDC-XM051609/2023	2023/5/20	23D	XBB.1.9.1	XBB.1.9.1	Omicron	23D (XBB.1.9)	GISAID	EPI_ISL_17729854
hCoV-19/Fujian/FCDC-XM051610/2023	2023/5/20	23D	XBB.1.9.1	XBB.1.9.1	Omicron	23D (XBB.1.9)	GISAID	EPI_ISL_17729855
hCoV-19/Fujian/FCDC-XM051611/2023	2023/5/20	22F	XBB.1.24.3	XBB.1.24.3	Omicron	22F (XBB)	GISAID	EPI_ISL_17729856
hCoV-19/Fujian/FCDC-XM051612/2023	2023/5/20	23D	XBB.1.9.1	XBB.1.9.1	Omicron	23D (XBB.1.9)	GISAID	EPI_ISL_17729857

hCoV-19/Fujian/FCDC-XM051613/2023	2023/5/20	23D	XBB.1.9.1	XBB.1.9.1	Omicron	23D (XBB.1.9)	GISAID	EPI_ISL_17729858
hCoV-19/Fujian/FCDC-XM052204/2023	2023/5/20	22F	XBB.1.42.2	XBB.1.42.2	Omicron	22F (XBB)	GISAID	EPI_ISL_17729859
hCoV-19/Fujian/FCDC-XM052205/2023	2023/5/20	23D	XBB.1.9.1	XBB.1.9.1	Omicron	23D (XBB.1.9)	GISAID	EPI_ISL_17729860
hCoV-19/Fujian/FCDC-XM052206/2023	2023/5/20	23B	XBB.1.16.18	XBB.1.16.18	Omicron	23B (XBB.1.16)	GISAID	EPI_ISL_17729861
hCoV-19/Fujian/FCDC-XM052207/2023	2023/5/20	23A	XBB.1.5	XBB.1.5	Omicron	23A (XBB.1.5)	GISAID	EPI_ISL_17729862
hCoV-19/Fujian/FCDC-XM052208/2023	2023/5/20	23F	EG.5.1.1	XBB.1.9.2.5.1.1	Omicron	23F (EG.5.1)	GISAID	EPI_ISL_17729863
hCoV-19/Fujian/FCDC-XM052209/2023	2023/5/20	23B	FU.1	XBB.1.16.1.1	Omicron	23B (XBB.1.16)	GISAID	EPI_ISL_17729864
hCoV-19/Fujian/FCDC-XM052210/2023	2023/5/20	23D	XBB.1.9.1	XBB.1.9.1	Omicron	23D (XBB.1.9)	GISAID	EPI_ISL_17729865
hCoV-19/Fujian/FCDC-	2023/5/20	23D	FL.2.3	XBB.1.9.1.2.3	Omicron	23D (XBB.1.9)	GISAID	EPI_ISL_17729866

XM052211/2023								
hCoV-19/Fujian/FCDC-XM052212/2023	2023/5/20	23D	XBB.1.9.1	XBB.1.9.1	Omicron	23D (XBB.1.9)	GISAID	EPI_ISL_17729867
hCoV-19/Fujian/FCDC-XM052213/2023	2023/5/20	23B	FU.1	XBB.1.16.1.1	Omicron	23B (XBB.1.16)	GISAID	EPI_ISL_17729869
hCoV-19/Fujian/FCDC-XM052214/2023	2023/5/20	23D	FL.2.3	XBB.1.9.1.2.3	Omicron	23D (XBB.1.9)	GISAID	EPI_ISL_17729870
hCoV-19/Fujian/FCDC-XM051512-2/2023	2023/5/21	23D	XBB.1.9.1	XBB.1.9.1	Omicron	23D (XBB.1.9)	GISAID	EPI_ISL_17729871
hCoV-19/Fujian/FCDC-XM051513-2/2023	2023/5/21	23D	FL.4	XBB.1.9.1.4	Omicron	23D (XBB.1.9)	GISAID	EPI_ISL_17729872
hCoV-19/Fujian/FCDC-XM051514-2/2023	2023/5/21	23B	XBB.1.16	XBB.1.16	Omicron	23B (XBB.1.16)	GISAID	EPI_ISL_17729873
hCoV-19/Fujian/FCDC-XM051517-2/2023	2023/5/21	23D	FL.13.1	XBB.1.9.1.13.1	Omicron	23D (XBB.1.9)	GISAID	EPI_ISL_17729874
hCoV-19/Fujian/FCDC-XM051520-2/2023	2023/5/21	23B	XBB.1.16.18	XBB.1.16.18	Omicron	23B (XBB.1.16)	GISAID	EPI_ISL_17729875
hCoV-19/Fujian/FC	2023/5/21	23D	FL.2.3	XBB.1.9.1.2.3	Omicron	23D (XBB.1.9)	GISAID	EPI_ISL_17729876

DC-XM051521-2/2023								
hCoV-19/Fujian/FCDC-XM051522-2/2023	2023/5/21	22F	XBB.1.31.2	XBB.1.31.2	Omicron	22F (XBB)	GISAID	EPI_ISL_17729877
hCoV-19/Fujian/FCDC-XM051523-2/2023	2023/5/21	23D	FL.21.2	XBB.1.9.1.21.2	Omicron	23D (XBB.1.9)	GISAID	EPI_ISL_17729878
hCoV-19/Fujian/FCDC-XM051524-2/2023	2023/5/21	23D	XBB.1.9.1	XBB.1.9.1	Omicron	23D (XBB.1.9)	GISAID	EPI_ISL_17729879
hCoV-19/Fujian/FCDC-XM052001/2023	2023/5/21	23D	XBB.1.9.1	XBB.1.9.1	Omicron	23D (XBB.1.9)	GISAID	EPI_ISL_17729880
hCoV-19/Fujian/FCDC-XM051625/2023	2023/5/25	23D	XBB.1.9.1	XBB.1.9.1	Omicron	23D (XBB.1.9)	GISAID	EPI_ISL_17801975
hCoV-19/Fujian/FCDC-XM051626/2023	2023/5/25	23A	XBB.1.5.15	XBB.1.5.15	Omicron	23A (XBB.1.5)	GISAID	EPI_ISL_17801976
hCoV-19/Fujian/FCDC-XM051627/2023	2023/5/25	23D	XBB.1.9.1	XBB.1.9.1	Omicron	23D (XBB.1.9)	GISAID	EPI_ISL_17801977
hCoV-19/Fujian/FCDC-XM051628/2023	2023/5/25	23D	XBB.1.9.1	XBB.1.9.1	Omicron	23D (XBB.1.9)	GISAID	EPI_ISL_17801978

hCoV-19/Fujian/FCDC-XM051629/2023	2023/5/25	23D	XBB.1.9.1	XBB.1.9.1	Omicron	23D (XBB.1.9)	GISAID	EPI_ISL_17801979
hCoV-19/Fujian/FCDC-XM051630/2023	2023/5/25	23D	FL.21.2	XBB.1.9.1.21.2	Omicron	23D (XBB.1.9)	GISAID	EPI_ISL_17801980
hCoV-19/Fujian/FCDC-XM051634/2023	2023/5/25	23D	FL.4.2	XBB.1.9.1.4.2	Omicron	23D (XBB.1.9)	GISAID	EPI_ISL_17801981
hCoV-19/Fujian/FCDC-XM051635/2023	2023/5/25	23D	XBB.1.9.1	XBB.1.9.1	Omicron	23D (XBB.1.9)	GISAID	EPI_ISL_17801982
hCoV-19/Fujian/FCDC-XM051636/2023	2023/5/25	23D	XBB.1.9.1	XBB.1.9.1	Omicron	23D (XBB.1.9)	GISAID	EPI_ISL_17801983
hCoV-19/Fujian/FCDC-XM051637/2023	2023/5/25	23D	XBB.1.9.1	XBB.1.9.1	Omicron	23D (XBB.1.9)	GISAID	EPI_ISL_17801984
hCoV-19/Fujian/FCDC-XM051638/2023	2023/5/25	23A	GF.1	XBB.1.5.24.1	Omicron	23A (XBB.1.5)	GISAID	EPI_ISL_17801985
hCoV-19/Fujian/FCDC-XM051639/2023	2023/5/25	23D	XBB.1.9.1	XBB.1.9.1	Omicron	23D (XBB.1.9)	GISAID	EPI_ISL_17801986
hCoV-19/Fujian/FCDC-	2023/6/2	23B	XBB.1.16.18	XBB.1.16.18	Omicron	23B (XBB.1.16)	GISAID	EPI_ISL_17801987

XM052302/2023								
hCoV-19/Fujian/FCDC-XM052303/2023	2023/6/2	23B	FU.1	XBB.1.16.1.1	Omicron	23B (XBB.1.16)	GISAID	EPI_ISL_17801988
hCoV-19/Fujian/FCDC-XM052304/2023	2023/6/2	23D	FL.21.2	XBB.1.9.1.21.2	Omicron	23D (XBB.1.9)	GISAID	EPI_ISL_17801989
hCoV-19/Fujian/FCDC-XM052305/2023	2023/6/2	23D	FL.2.3	XBB.1.9.1.2.3	Omicron	23D (XBB.1.9)	GISAID	EPI_ISL_17801990
hCoV-19/Fujian/FCDC-XM052306/2023	2023/6/2	23D	XBB.1.9.1	XBB.1.9.1	Omicron	23D (XBB.1.9)	GISAID	EPI_ISL_17801991
hCoV-19/Fujian/FCDC-XM052307/2023	2023/6/2	23D	XBB.1.9.1	XBB.1.9.1	Omicron	23D (XBB.1.9)	GISAID	EPI_ISL_17801992
hCoV-19/Fujian/FCDC-XM052309/2023	2023/6/2	23D	XBB.1.9.1	XBB.1.9.1	Omicron	23D (XBB.1.9)	GISAID	EPI_ISL_17801993
hCoV-19/Fujian/FCDC-XM052310/2023	2023/6/2	23D	XBB.1.9.1	XBB.1.9.1	Omicron	23D (XBB.1.9)	GISAID	EPI_ISL_17801994
hCoV-19/Fujian/FCDC-XM052311/2023	2023/6/2	23A	GB.1	XBB.1.5.46.1	Omicron	23A (XBB.1.5)	GISAID	EPI_ISL_17801995
hCoV-19/Fujian/FC	2023/6/2	23D	XBB.1.9.1	XBB.1.9.1	Omicron	23D (XBB.1.9)	GISAID	EPI_ISL_17801996

DC-XM052312/2023								
hCoV-19/Fujian/FCDC-XM060501/2023	2023/6/5	23D	XBB.1.9.1	XBB.1.9.1	Omicron	23D (XBB.1.9)	GISAID	EPI_ISL_17802010
hCoV-19/Fujian/FCDC-XM052321/2023	2023/6/9	23B	XBB.1.16.1	XBB.1.16.1	Omicron	23B (XBB.1.16)	GISAID	EPI_ISL_17819887
hCoV-19/Fujian/FCDC-XM052316/2023	2023/6/9	23B	XBB.1.16	XBB.1.16	Omicron	23B (XBB.1.16)	GISAID	EPI_ISL_17819888
hCoV-19/Fujian/FCDC-XM052313/2023	2023/6/9	23D	XBB.1.9.1	XBB.1.9.1	Omicron	23D (XBB.1.9)	GISAID	EPI_ISL_17819889
hCoV-19/Fujian/FCDC-XM052319/2023	2023/6/9	23A	GF.1	XBB.1.5.24.1	Omicron	23A (XBB.1.5)	GISAID	EPI_ISL_17819890
hCoV-19/Fujian/FCDC-XM052323/2023	2023/6/9	23D	XBB.1.9.1	XBB.1.9.1	Omicron	23D (XBB.1.9)	GISAID	EPI_ISL_17819891
hCoV-19/Fujian/FCDC-XM052322/2023	2023/6/9	22F	FY.3.1	XBB.1.22.1.3.1	Omicron	22F (XBB)	GISAID	EPI_ISL_17819892
hCoV-19/Fujian/FCDC-XM052314/2023	2023/6/9	23D	FL.2.3	XBB.1.9.1.2.3	Omicron	23D (XBB.1.9)	GISAID	EPI_ISL_17819893

hCoV-19/Fujian/FCDC-XM061509/2023	2023/6/15	23F	EG.5.1.1	XBB.1.9.2.5.1.1	Omicron	23F (EG.5.1)	GISAID	EPI_ISL_17850211
hCoV-19/Fujian/FCDC-XM061510/2023	2023/6/15	23D	FL.4.11	XBB.1.9.1.4.11	Omicron	23D (XBB.1.9)	GISAID	EPI_ISL_17850212
hCoV-19/Fujian/FCDC-XM061511/2023	2023/6/15	23F	EG.5.1.1	XBB.1.9.2.5.1.1	Omicron	23F (EG.5.1)	GISAID	EPI_ISL_17850213
hCoV-19/Fujian/FCDC-XM061512/2023	2023/6/15	23F	EG.5.1.1	XBB.1.9.2.5.1.1	Omicron	23F (EG.5.1)	GISAID	EPI_ISL_17850214
hCoV-19/Fujian/FCDC-XM061513/2023	2023/6/15	23B	FU.1	XBB.1.16.1.1	Omicron	23B (XBB.1.16)	GISAID	EPI_ISL_17850215
hCoV-19/Fujian/FCDC-XM061514/2023	2023/6/15	23D	FL.13.2	XBB.1.9.1.13.2	Omicron	23D (XBB.1.9)	GISAID	EPI_ISL_17850216
hCoV-19/Fujian/FCDC-XM061515/2023	2023/6/15	23D	FL.13.2	XBB.1.9.1.13.2	Omicron	23D (XBB.1.9)	GISAID	EPI_ISL_17850217
hCoV-19/Fujian/FCDC-XM061516/2023	2023/6/15	23D	FL.2.3	XBB.1.9.1.2.3	Omicron	23D (XBB.1.9)	GISAID	EPI_ISL_17850218
hCoV-19/Fujian/FCDC-	2023/6/15	23D	FL.2.3	XBB.1.9.1.2.3	Omicron	23D (XBB.1.9)	GISAID	EPI_ISL_17850219

XM061517/2023								
hCoV-19/Fujian/FCDC-XM061518/2023	2023/6/15	23D	XBB.1.9.1	XBB.1.9.1	Omicron	23D (XBB.1.9)	GISAID	EPI_ISL_17850220
hCoV-19/Fujian/FCDC-XM061519/2023	2023/6/15	23D	XBB.1.9.1	XBB.1.9.1	Omicron	23D (XBB.1.9)	GISAID	EPI_ISL_17850221
hCoV-19/Fujian/FCDC-XM061520/2023	2023/6/15	23A	GF.1	XBB.1.5.24.1	Omicron	23A (XBB.1.5)	GISAID	EPI_ISL_17850222
hCoV-19/Fujian/FCDC-XM061521/2023	2023/6/21	23F	EG.5.1.1	XBB.1.9.2.5.1.1	Omicron	23F (EG.5.1)	GISAID	EPI_ISL_17956738
hCoV-19/Fujian/FCDC-XM061522/2023	2023/6/21	23B	FU.1	XBB.1.16.1.1	Omicron	23B (XBB.1.16)	GISAID	EPI_ISL_17956739
hCoV-19/Fujian/FCDC-XM061523/2023	2023/6/21	23F	EG.5.1.1	XBB.1.9.2.5.1.1	Omicron	23F (EG.5.1)	GISAID	EPI_ISL_17956740
hCoV-19/Fujian/FCDC-XM061524/2023	2023/6/21	23D	XBB.1.9.1	XBB.1.9.1	Omicron	23D (XBB.1.9)	GISAID	EPI_ISL_17956741
hCoV-19/Fujian/FCDC-XM061525/2023	2023/6/21	23D	XBB.1.9.1	XBB.1.9.1	Omicron	23D (XBB.1.9)	GISAID	EPI_ISL_17956742
hCoV-19/Fujian/FC	2023/6/21	23D	XBB.1.9.1	XBB.1.9.1	Omicron	23D (XBB.1.9)	GISAID	EPI_ISL_17956743

DC-XM061526/2023								
hCoV-19/Fujian/FCDC-XM061527/2023	2023/6/21	23D	FL.10	XBB.1.9.1.10	Omicron	23D (XBB.1.9)	GISAID	EPI_ISL_17956744
hCoV-19/Fujian/FCDC-XM061528/2023	2023/6/21	23B	GY.1	XBB.1.16.2.1	Omicron	23B (XBB.1.16)	GISAID	EPI_ISL_17956745
hCoV-19/Fujian/FCDC-XM061529/2023	2023/6/21	23D	FL.16	XBB.1.9.1.16	Omicron	23D (XBB.1.9)	GISAID	EPI_ISL_17956746
hCoV-19/Fujian/FCDC-XM061530/2023	2023/6/21	22D	FR.1.3	BA.2.75.5.1.2.3.1.3	Omicron	22D (BA.2.75)	GISAID	EPI_ISL_17956747
hCoV-19/Fujian/FCDC-XM061531/2023	2023/6/21	23B	FU.1	XBB.1.16.1.1	Omicron	23B (XBB.1.16)	GISAID	EPI_ISL_17956748
hCoV-19/Fujian/FCDC-XM061532/2023	2023/6/21	23B	XBB.1.16.18	XBB.1.16.18	Omicron	23B (XBB.1.16)	GISAID	EPI_ISL_17956749
hCoV-19/Fujian/FCDC-XM071201/2023	2023/7/7	23D	FL.4	XBB.1.9.1.4	Omicron	23D (XBB.1.9)	GISAID	EPI_ISL_17999101
hCoV-19/Fujian/FCDC-XM071202/2023	2023/7/7	23B	GY.1	XBB.1.16.2.1	Omicron	23B (XBB.1.16)	GISAID	EPI_ISL_17999102

hCoV-19/Fujian/FCDC-XM071203/2023	2023/7/7	23D	FL.13.2	XBB.1.9.1.13.2	Omicron	23D (XBB.1.9)	GISAID	EPI_ISL_17999103
hCoV-19/Fujian/FCDC-XM071204/2023	2023/7/7	23D	FL.4	XBB.1.9.1.4	Omicron	23D (XBB.1.9)	GISAID	EPI_ISL_17999104
hCoV-19/Fujian/FCDC-XM071205/2023	2023/7/7	23F	EG.5.1.1	XBB.1.9.2.5.1.1	Omicron	23F (EG.5.1)	GISAID	EPI_ISL_17999105
hCoV-19/Fujian/FCDC-XM071206/2023	2023/7/7	23B	XBB.1.16	XBB.1.16	Omicron	23B (XBB.1.16)	GISAID	EPI_ISL_17999106
hCoV-19/Fujian/FCDC-XM071208/2023	2023/7/7	23A	GF.1	XBB.1.5.24.1	Omicron	23A (XBB.1.5)	GISAID	EPI_ISL_17999107
hCoV-19/Fujian/FCDC-XM071209/2023	2023/7/7	23B	FU.1	XBB.1.16.1.1	Omicron	23B (XBB.1.16)	GISAID	EPI_ISL_17999108
hCoV-19/Fujian/FCDC-XM071210/2023	2023/7/7	23D	FL.16	XBB.1.9.1.16	Omicron	23D (XBB.1.9)	GISAID	EPI_ISL_17999109
hCoV-19/Fujian/FCDC-XM071211/2023	2023/7/7	23D	XBB.1.9.1	XBB.1.9.1	Omicron	23D (XBB.1.9)	GISAID	EPI_ISL_17999110
hCoV-19/Fujian/FCDC-	2023/7/7	23F	EG.5.1.1	XBB.1.9.2.5.1.1	Omicron	23F (EG.5.1)	GISAID	EPI_ISL_17999111

XM071212/2023								
hCoV-19/Fujian/FCDC-XM071213/2023	2023/7/7	22F	FY.3.2	XBB.1.22.1.3.2	Omicron	22F (XBB)	GISAID	EPI_ISL_17999112
hCoV-19/Fujian/FCDC-XM071214/2023	2023/7/7	23F	EG.5.1.1	XBB.1.9.2.5.1.1	Omicron	23F (EG.5.1)	GISAID	EPI_ISL_17999113
hCoV-19/Fujian/FCDC-XM071215/2023	2023/7/7	23B	XBB.1.16.18	XBB.1.16.18	Omicron	23B (XBB.1.16)	GISAID	EPI_ISL_17999114
hCoV-19/Fujian/FCDC-XM080201/2023	2023/7/28	23B	GY.1	XBB.1.16.2.1	Omicron	23B (XBB.1.16)	GISAID	EPI_ISL_18078177
hCoV-19/Fujian/FCDC-XM080202/2023	2023/7/28	23F	EG.5.1.1	XBB.1.9.2.5.1.1	Omicron	23F (EG.5.1)	GISAID	EPI_ISL_18078178
hCoV-19/Fujian/FCDC-XM080203/2023	2023/7/28	23D	FL.13.2	XBB.1.9.1.13.2	Omicron	23D (XBB.1.9)	GISAID	EPI_ISL_18078179
hCoV-19/Fujian/FCDC-XM080204/2023	2023/7/28	23D	FL.2.3	XBB.1.9.1.2.3	Omicron	23D (XBB.1.9)	GISAID	EPI_ISL_18078180
hCoV-19/Fujian/FCDC-XM080206/2023	2023/7/28	23B	FU.2.1	XBB.1.16.1.2.1	Omicron	23B (XBB.1.16)	GISAID	EPI_ISL_18108896
hCoV-19/Fujian/FC	2023/7/28	22F	FY.3.1	XBB.1.22.1.3.1	Omicron	22F (XBB)	GISAID	EPI_ISL_18108897

DC-XM080207/2023								
hCoV-19/Fujian/FCDC-XM080208/2023	2023/7/28	22F	FE.1.1	XBB.1.18.1.1.1	Omicron	22F (XBB)	GISAID	EPI_ISL_18108898
hCoV-19/Fujian/FCDC-XM080213/2023	2023/7/28	23E	GJ.3	XBB.2.3.3.3	Omicron	23E (XBB.2.3)	GISAID	EPI_ISL_18127173
hCoV-19/Fujian/FCDC-XM080214/2023	2023/7/28	22F	FY.3.1	XBB.1.22.1.3.1	Omicron	22F (XBB)	GISAID	EPI_ISL_18127174
hCoV-19/Fujian/FCDC-XM080209/2023	2023/8/12	23F	EG.5.1.1	XBB.1.9.2.5.1.1	Omicron	23F (EG.5.1)	GISAID	EPI_ISL_18127175
hCoV-19/Fujian/FCDC-XM080210/2023	2023/8/12	23F	EG.5.1.1	XBB.1.9.2.5.1.1	Omicron	23F (EG.5.1)	GISAID	EPI_ISL_18127176
hCoV-19/Fujian/FCDC-XM080211/2023	2023/8/12	23D	XBB.1.9.1	XBB.1.9.1	Omicron	23D (XBB.1.9)	GISAID	EPI_ISL_18145965
hCoV-19/Fujian/FCDC-XM080212/2023	2023/8/12	23D	FL.2.3	XBB.1.9.1.2.3	Omicron	23D (XBB.1.9)	GISAID	EPI_ISL_18145966
hCoV-19/Fujian/FCDC-XM080216/2023	2023/8/24	23D	FL.13.2	XBB.1.9.1.13.2	Omicron	23D (XBB.1.9)	GISAID	EPI_ISL_18213249

hCoV-19/Fujian/FCDC-XM080215/2023	2023/8/24	23F	EG.5.1.1	XBB.1.9.2.5.1.1	Omicron	23F (EG.5.1)	GISAID	EPI_ISL_18213250

Table.3 SARS-CoV-2 reference sequences downloaded from GSAID

name	date	clade	Nextclade_pango	partiallyAliased	clade_who	clade_display	origin	gisaid_epi_isl
hCoV-19/Singapore/R72MR174/2023	2023/10/2	23F	EG.5.1.8	XBB.1.9.2.5.1.8	Omicron	23F (EG.5.1)	reference	EPI_ISL_18362115
hCoV-19/Singapore/R72MR12/2023	2023/10/2	23E	XBB.2.3	XBB.2.3	Omicron	23E (XBB.2.3)	reference	EPI_ISL_18361990
hCoV-19/USA/OR-OHSU-232290056/2023	2023/5/15	23D	FL.4	XBB.1.9.1.4	Omicron	23D (XBB.1.9)	reference	EPI_ISL_18342687
hCoV-19/Singapore/R71MR117/2023	2023/9/28	23B	XBB.1.16.17	XBB.1.16.17	Omicron	23B (XBB.1.16)	reference	EPI_ISL_18361821
hCoV-19/Singapore/R72MR32/2023	2023/10/2	23B	XBB.1.16	XBB.1.16	Omicron	23B (XBB.1.16)	reference	EPI_ISL_18362058
hCoV-19/England/WSI-33113304/2023	2023/9/20	23B	XBB.1.16	XBB.1.16	Omicron	23B (XBB.1.16)	reference	EPI_ISL_18362157
hCoV-19/CanaryIslands/HUGCDN-04270267/2023	2023/9/9	23A	XBB.1.5.103	XBB.1.5.103	Omicron	23A (XBB.1.5)	reference	EPI_ISL_18361422
hCoV-19/Spain/GA-HUAC-11483/2023	2023/9/29	23A	JD.1.1	XBB.1.5.102.1.1	Omicron	23A (XBB.1.5)	reference	EPI_ISL_18361492
hCoV-19/Singapore/R71MR40/2023	2023/9/28	23B	XBB.1.16	XBB.1.16	Omicron	23B (XBB.1.16)	reference	EPI_ISL_18361846
hCoV-19/USA/MI-SHMSU-A12_I_0106_65/2022	2022/11/14	22F	XBB	XBB	Omicron	22F (XBB)	reference	EPI_ISL_18335854
hCoV-19/USA/MI-SHMSU-F09_I_1130_68/2022	2022/11/28	22F	XBB	XBB	Omicron	22F (XBB)	reference	EPI_ISL_18336517
hCoV-19/Cameroon/V-10519/2022	2022/3/12	22E	BQ.1	BA.5.3.1.1.1.1.1	Omicron	22E (BQ.1)	reference	EPI_ISL_18352124

hCoV-19/Sweden/C-03_SE600_500000204675SP/2022	2022/11/15	22E	BQ.1	BA.5.3.1.1.1.1.1	Omicron	22E (BQ.1)	reference	EPI_ISL_18359936
hCoV-19/env/India/MH-FMR_K4-8a/2022	2022/9/12	22D	BY.1	BA.2.75.6.1	Omicron	22D (BA.2.75)	reference	EPI_ISL_18226310
hCoV-19/Russia/MOW-RII-MH143891S/2023	2023/4/11	22D	BY.1	BA.2.75.6.1	Omicron	22D (BA.2.75)	reference	EPI_ISL_18238446
hCoV-19/env/Malaysia/UMS-W110805/2023	2023/8/11	22B	BA.5	BA.5	Omicron	22B (BA.5)	reference	EPI_ISL_1833447
hCoV-19/Cameroon/V-06281/2022	2022/2/4	22A	BA.4	BA.4	Omicron	22A (BA.4)	reference	EPI_ISL_18352132
hCoV-19/Denmark/DCGC-660209/2022	2022/6/27	22A	BA.4	BA.4	Omicron	22A (BA.4)	reference	EPI_ISL_18353012
hCoV-19/Denmark/DCGC-660666/2022	2022/8/1	22A	BA.4	BA.4	Omicron	22A (BA.4)	reference	EPI_ISL_18353447
hCoV-19/England/WSI-3311339B/2022	2022/4/7	21L	BA.2	BA.2	Omicron	21L (BA.2)	reference	EPI_ISL_18362135
hCoV-19/SouthAfrica/NHLS-UCT-GS-BC36/2022	2022/1/10	21K	BA.1.18	BA.1.18	Omicron	21K (BA.1)	reference	EPI_ISL_18300512
hCoV-19/SouthAfrica/NHLS-UCT-GS-BE65/2021	2021/12/22	21K	BA.1.17	BA.1.17	Omicron	21K (BA.1)	reference	EPI_ISL_18300513
hCoV-19/Denmark/DCGC-660612/2022	2022/2/7	21K	BA.1	BA.1	Omicron	21K (BA.1)	reference	EPI_ISL_18353397
hCoV-19/HongKong/HKU-182/2021	2021/4/18	21B	B.1.617.1	B.1.617.1	Kappa	21B (Kappa)	reference	EPI_ISL_16347666
hCoV-19/HongKong/HKU-184/2021	2021/4/28	21B	B.1.617.1	B.1.617.1	Kappa	21B (Kappa)	reference	EPI_ISL_16347668
hCoV-19/India/MP-AIIMS_B-2352/2022	2022/1/31	21B	B.1.617.1	B.1.617.1	Kappa	21B (Kappa)	reference	EPI_ISL_18004955
hCoV-19/Denmark/DCGC-660515/2021	2021/11/8	21J	B.1.617.2	B.1.617.2	Delta	21J (Delta)	reference	EPI_ISL_18353304
hCoV-19/CzechRepublic/UMTM251044/2021	2021/8/17	21J	B.1.617.2	B.1.617.2	Delta	21J (Delta)	reference	EPI_ISL_18358387
hCoV-19/SouthAfrica/NHLS-UCT-GP-K050/2021	2021/6/3	20H	B.1.351	B.1.351	Beta	20H (Beta)	reference	EPI_ISL_18340704
hCoV-19/Malaysia/MGVI_UBM21023517/2021	2021/7/26	20H	B.1.351	B.1.351	Beta	20H (Beta)	reference	EPI_ISL_18346961
hCoV-19/Wuhan/WIV06/2019	2019/12/30	19A	B	B		19A	reference	EPI_ISL_402129
hCoV-19/Wuhan/HBCDC-HB-	2019/1	19A	B	B		19A	refer	EPI_ISL_4

01/2019	2/30						ence	02132
hCoV-19/England/PHEC-YY8ME8P/2022	2022/11/10	23C	CH.1.1	BA.2.75.3.4.1.1.1.1	Omicron	23C (CH.1.1)	reference	EPI_ISL_15726171
hCoV-19/England/PHEC-YY8OBMB/2022	2022/11/11	23C	CH.1.1	BA.2.75.3.4.1.1.1.1	Omicron	23C (CH.1.1)	reference	EPI_ISL_16074954
hCoV-19/USA/OH-PLMI-HCF7G-16004/2022	2022/12/12	23C	CH.1.1	BA.2.75.3.4.1.1.1.1	Omicron	23C (CH.1.1)	reference	EPI_ISL_16448854
